# Supplementary material for: Resolving the full spectrum of human genome variation using Linked-Reads
Source: Genome Res. 2019 Apr;29(4):635–45. doi: 10.1101/gr.234443.118 (PMC6442396; doi:10.1101/gr.234443.118)
Supplement: Supplemental Material [file supp_gr.234443.118_Supplemental_longranger-2.2.zip › longranger-2.2.tar.bz2!/lr2.2-public/lib/stan/cmdstan/stan/doc/api/html/namespacestan_1_1common.html]

Stan: stan::common Namespace Reference


|  |  |
| --- | --- |
|  | Stan  2.5.0  probability, sampling & optimization |


- Main Page
- Related Pages
- Namespaces
- Classes
- Files

- Namespace List
- Namespace Members

All Classes Namespaces Files Functions Variables Typedefs Enumerations Enumerator Friends Macros Pages

- stan
- common

Namespaces |
Classes |
Functions

stan::common Namespace Reference

|  |  |
| --- | --- |
| Namespaces | |
|  | recorder |
|  | |

|  |  |
| --- | --- |
| Classes | |
| struct | NoOpFunctor |
|  | |
| class | var\_context\_factory |
|  | |
| class | dump\_factory |
|  | |

|  |  |
| --- | --- |
| Functions | |
| template<class Model > | |
| int | command (int argc, const char \*argv[]) |
|  | |
| template<typename ModelT , typename BFGSOptimizerT , typename RNGT , typename StartIterationCallback > | |
| int | do\_bfgs\_optimize (ModelT &model, BFGSOptimizerT &bfgs, RNGT &base\_rng, double &lp, std::vector< double > &cont\_vector, std::vector< int > &disc\_vector, std::fstream \*output\_stream, std::ostream \*notice\_stream, bool save\_iterations, int refresh, StartIterationCallback &callback) |
|  | |
| bool | do\_print (const int n, const bool special, const int refresh) |
|  | Indicates whether it should print on the current iteration. More... |
|  | |
| bool | do\_print (const int n, const int refresh) |
|  | Indicates whether it should print on the current iteration. More... |
|  | |
| template<class Sampler > | |
| bool | init\_adapt (Sampler \*sampler, const double delta, const double gamma, const double kappa, const double t0, const Eigen::VectorXd &cont\_params) |
|  | |
| template<class Sampler > | |
| bool | init\_adapt (stan::mcmc::base\_mcmc \*sampler, stan::gm::categorical\_argument \*adapt, const Eigen::VectorXd &cont\_params) |
|  | |
| template<class Sampler > | |
| bool | init\_nuts (stan::mcmc::base\_mcmc \*sampler, stan::gm::argument \*algorithm) |
|  | |
| template<class Sampler > | |
| bool | init\_static\_hmc (stan::mcmc::base\_mcmc \*sampler, stan::gm::argument \*algorithm) |
|  | |
| template<class Sampler > | |
| bool | init\_windowed\_adapt (stan::mcmc::base\_mcmc \*sampler, stan::gm::categorical\_argument \*adapt, unsigned int num\_warmup, const Eigen::VectorXd &cont\_params) |
|  | |
| template<class Model > | |
| bool | initialize\_state\_zero (Eigen::VectorXd &cont\_params, Model &model, std::ostream \*output) |
|  | Sets initial state to zero. More... |
|  | |
| template<class Model , class RNG > | |
| bool | initialize\_state\_random (const double R, Eigen::VectorXd &cont\_params, Model &model, RNG &base\_rng, std::ostream \*output) |
|  | Initializes state to random uniform values within range. More... |
|  | |
| template<class ContextFactory , class Model , class RNG > | |
| bool | initialize\_state\_source (const std::string source, Eigen::VectorXd &cont\_params, Model &model, RNG &base\_rng, std::ostream \*output, ContextFactory &context\_factory) |
|  | Creates the initial state using the source parameter. More... |
|  | |
| bool | get\_double\_from\_string (const std::string &s, double &val) |
|  | Converts string to double. More... |
|  | |
| template<class ContextFactory , class Model , class RNG > | |
| bool | initialize\_state (const std::string init, Eigen::VectorXd &cont\_params, Model &model, RNG &base\_rng, std::ostream \*output, ContextFactory &context\_factory) |
|  | Creates the initial state. More... |
|  | |
| void | print\_progress (const int m, const int start, const int finish, const int refresh, const bool warmup, const std::string prefix, const std::string suffix, std::ostream &o) |
|  | |
| template<class Model , class RNG , class StartTransitionCallback , class SampleRecorder , class DiagnosticRecorder , class MessageRecorder > | |
| void | run\_markov\_chain (stan::mcmc::base\_mcmc \*sampler, const int num\_iterations, const int start, const int finish, const int num\_thin, const int refresh, const bool save, const bool warmup, stan::io::mcmc\_writer< Model, SampleRecorder, DiagnosticRecorder, MessageRecorder > &writer, stan::mcmc::sample &init\_s, Model &model, RNG &base\_rng, const std::string &prefix, const std::string &suffix, std::ostream &o, StartTransitionCallback &callback) |
|  | |
| template<class Model , class RNG , class StartTransitionCallback , class SampleRecorder , class DiagnosticRecorder , class MessageRecorder > | |
| void | sample (stan::mcmc::base\_mcmc \*sampler, int num\_warmup, int num\_samples, int num\_thin, int refresh, bool save, stan::io::mcmc\_writer< Model, SampleRecorder, DiagnosticRecorder, MessageRecorder > &writer, stan::mcmc::sample &init\_s, Model &model, RNG &base\_rng, const std::string &prefix, const std::string &suffix, std::ostream &o, StartTransitionCallback &callback) |
|  | |
| template<class Model , class RNG , class StartTransitionCallback , class SampleRecorder , class DiagnosticRecorder , class MessageRecorder > | |
| void | warmup (stan::mcmc::base\_mcmc \*sampler, int num\_warmup, int num\_samples, int num\_thin, int refresh, bool save, stan::io::mcmc\_writer< Model, SampleRecorder, DiagnosticRecorder, MessageRecorder > &writer, stan::mcmc::sample &init\_s, Model &model, RNG &base\_rng, const std::string &prefix, const std::string &suffix, std::ostream &o, StartTransitionCallback &callback) |
|  | |
| void | write\_error\_msg (std::ostream \*error\_stream, const std::exception &e) |
|  | |
| template<class Model , class RNG > | |
| void | write\_iteration (std::ostream &output\_stream, Model &model, RNG &base\_rng, double lp, std::vector< double > &cont\_vector, std::vector< int > &disc\_vector) |
|  | |
| void | write\_iteration\_csv (std::ostream &output\_stream, const double lp, const std::vector< double > &model\_values) |
|  | |
| void | write\_model (std::ostream \*s, const std::string model\_name, const std::string prefix="") |
|  | |
| void | write\_stan (std::ostream \*s, const std::string prefix="") |
|  | |

## Function Documentation

template<class Model >

|  |  |  |  |
| --- | --- | --- | --- |
| int stan::common::command | ( | int | *argc*, |
|  |  | const char \* | *argv*[] |
|  | ) |  |  |

Definition at line 67 of file command.hpp.

template<typename ModelT , typename BFGSOptimizerT , typename RNGT , typename StartIterationCallback >

|  |  |  |  |
| --- | --- | --- | --- |
| int stan::common::do\_bfgs\_optimize | ( | ModelT & | *model*, |
|  |  | BFGSOptimizerT & | *bfgs*, |
|  |  | RNGT & | *base\_rng*, |
|  |  | double & | *lp*, |
|  |  | std::vector< double > & | *cont\_vector*, |
|  |  | std::vector< int > & | *disc\_vector*, |
|  |  | std::fstream \* | *output\_stream*, |
|  |  | std::ostream \* | *notice\_stream*, |
|  |  | bool | *save\_iterations*, |
|  |  | int | *refresh*, |
|  |  | StartIterationCallback & | *callback* |
|  | ) |  |  |

Definition at line 21 of file do\_bfgs\_optimize.hpp.

|  |  |  |  |
| --- | --- | --- | --- |
| bool stan::common::do\_print | ( | const int | *n*, |
|  |  | const bool | *special*, |
|  |  | const int | *refresh* |
|  | ) |  |  |

Indicates whether it should print on the current iteration.

The function returns: true if refresh > 0 and (n == 0 or (n + 1) % refresh == 0 or special == true) false otherwise. Examples: if refresh = 0, always returns false. if refresh = 10 and special == false, returns true for n = {0, 9, 19, ...}, returns false for n = {1 - 8, 10 - 18, 20 - 28, ...}. if refresh = 10 and special == true, returns true for all n.

Parameters
:   |  |  |
    | --- | --- |
    | n | Iteration number |
    | special | When true, returns true |
    | refresh | Number of iterations to refresh |

Definition at line 25 of file do\_print.hpp.

|  |  |  |  |
| --- | --- | --- | --- |
| bool stan::common::do\_print | ( | const int | *n*, |
|  |  | const int | *refresh* |
|  | ) |  |  |

Indicates whether it should print on the current iteration.

The function returns: true if refresh > 0 and (n == 0 or (n + 1) % refresh == 0) false otherwise. Examples: if refresh = 0, always returns false. if refresh = 10, returns true for n = {0, 9, 19, ...}, returns false for n = {1 - 8, 10 - 18, 20 - 28, ...}.

Parameters
:   |  |  |
    | --- | --- |
    | n | Iteration number |
    | refresh | Number of iterations to refresh |

Definition at line 43 of file do\_print.hpp.

|  |  |  |  |
| --- | --- | --- | --- |
| bool stan::common::get\_double\_from\_string | ( | const std::string & | *s*, |
|  |  | double & | *val* |
|  | ) |  |  |

Converts string to double.

Returns true if it is able to convert the number, false otherwise.

Parameters
:   |  |  |  |
    | --- | --- | --- |
    | [in] | s | string input |
    | [out] | val | the double value of the string if it is parsable as a double; else NaN |

Definition at line 208 of file initialize\_state.hpp.

template<class Sampler >

|  |  |  |  |
| --- | --- | --- | --- |
| bool stan::common::init\_adapt | ( | Sampler \* | *sampler*, |
|  |  | const double | *delta*, |
|  |  | const double | *gamma*, |
|  |  | const double | *kappa*, |
|  |  | const double | *t0*, |
|  |  | const Eigen::VectorXd & | *cont\_params* |
|  | ) |  |  |

Definition at line 13 of file init\_adapt.hpp.

template<class Sampler >

|  |  |  |  |
| --- | --- | --- | --- |
| bool stan::common::init\_adapt | ( | stan::mcmc::base\_mcmc \* | *sampler*, |
|  |  | stan::gm::categorical\_argument \* | *adapt*, |
|  |  | const Eigen::VectorXd & | *cont\_params* |
|  | ) |  |  |

Definition at line 42 of file init\_adapt.hpp.

template<class Sampler >

|  |  |  |  |
| --- | --- | --- | --- |
| bool stan::common::init\_nuts | ( | stan::mcmc::base\_mcmc \* | *sampler*, |
|  |  | stan::gm::argument \* | *algorithm* |
|  | ) |  |  |

Definition at line 13 of file init\_nuts.hpp.

template<class Sampler >

|  |  |  |  |
| --- | --- | --- | --- |
| bool stan::common::init\_static\_hmc | ( | stan::mcmc::base\_mcmc \* | *sampler*, |
|  |  | stan::gm::argument \* | *algorithm* |
|  | ) |  |  |

Definition at line 13 of file init\_static\_hmc.hpp.

template<class Sampler >

|  |  |  |  |
| --- | --- | --- | --- |
| bool stan::common::init\_windowed\_adapt | ( | stan::mcmc::base\_mcmc \* | *sampler*, |
|  |  | stan::gm::categorical\_argument \* | *adapt*, |
|  |  | unsigned int | *num\_warmup*, |
|  |  | const Eigen::VectorXd & | *cont\_params* |
|  | ) |  |  |

Definition at line 13 of file init\_windowed\_adapt.hpp.

template<class ContextFactory , class Model , class RNG >

|  |  |  |  |
| --- | --- | --- | --- |
| bool stan::common::initialize\_state | ( | const std::string | *init*, |
|  |  | Eigen::VectorXd & | *cont\_params*, |
|  |  | Model & | *model*, |
|  |  | RNG & | *base\_rng*, |
|  |  | std::ostream \* | *output*, |
|  |  | ContextFactory & | *context\_factory* |
|  | ) |  |  |

Creates the initial state.

Parameters
:   |  |  |  |
    | --- | --- | --- |
    | [in] | init | init can either be "0", a number as a string, or a filename. |
    | [out] | cont\_params | the initialized state. This should be the right size and set to 0. |
    | [in,out] | model | the model. Side effects on model? I'm not quite sure |
    | [in,out] | base\_rng | the random number generator. State may change. |
    | [in,out] | output | output stream for messages |
    | [in,out] | context\_factory | an instantiated factory that implements the concept of a context\_factory. This has one method that takes a string. |

Definition at line 234 of file initialize\_state.hpp.

template<class Model , class RNG >

|  |  |  |  |
| --- | --- | --- | --- |
| bool stan::common::initialize\_state\_random | ( | const double | *R*, |
|  |  | Eigen::VectorXd & | *cont\_params*, |
|  |  | Model & | *model*, |
|  |  | RNG & | *base\_rng*, |
|  |  | std::ostream \* | *output* |
|  | ) |  |  |

Initializes state to random uniform values within range.

Parameters
:   |  |  |  |
    | --- | --- | --- |
    | [in] | R | valid range of the initialization; must be greater than or equal to 0. |
    | [out] | cont\_params | the initialized state. This should be the right size and set to 0. |
    | [in,out] | model | the model. Side effects on model? I'm not quite sure |
    | [in,out] | base\_rng | the random number generator. State may change. |
    | [in,out] | output | output stream for messages |

Definition at line 82 of file initialize\_state.hpp.

template<class ContextFactory , class Model , class RNG >

|  |  |  |  |
| --- | --- | --- | --- |
| bool stan::common::initialize\_state\_source | ( | const std::string | *source*, |
|  |  | Eigen::VectorXd & | *cont\_params*, |
|  |  | Model & | *model*, |
|  |  | RNG & | *base\_rng*, |
|  |  | std::ostream \* | *output*, |
|  |  | ContextFactory & | *context\_factory* |
|  | ) |  |  |

Creates the initial state using the source parameter.

Parameters
:   |  |  |  |
    | --- | --- | --- |
    | [in] | source | a string that the context\_factory can interpret and provide a valid var\_context |
    | [out] | cont\_params | the initialized state. This should be the right size and set to 0. |
    | [in,out] | model | the model. Side effects on model? I'm not quite sure |
    | [in,out] | base\_rng | the random number generator. State may change. |
    | [in,out] | output | output stream for messages |
    | [in,out] | context\_factory | an instantiated factory that implements the concept of a context\_factory. This has one method that takes a string. |

Definition at line 154 of file initialize\_state.hpp.

template<class Model >

|  |  |  |  |
| --- | --- | --- | --- |
| bool stan::common::initialize\_state\_zero | ( | Eigen::VectorXd & | *cont\_params*, |
|  |  | Model & | *model*, |
|  |  | std::ostream \* | *output* |
|  | ) |  |  |

Sets initial state to zero.

Parameters
:   |  |  |  |
    | --- | --- | --- |
    | [out] | cont\_params | the initialized state. This should be the right size and set to 0. |
    | [in,out] | model | the model. Side effects on model? I'm not quite sure |
    | [in,out] | output | output stream for messages |

Definition at line 33 of file initialize\_state.hpp.

|  |  |  |  |
| --- | --- | --- | --- |
| void stan::common::print\_progress | ( | const int | *m*, |
|  |  | const int | *start*, |
|  |  | const int | *finish*, |
|  |  | const int | *refresh*, |
|  |  | const bool | *warmup*, |
|  |  | const std::string | *prefix*, |
|  |  | const std::string | *suffix*, |
|  |  | std::ostream & | *o* |
|  | ) |  |  |

Definition at line 13 of file print\_progress.hpp.

template<class Model , class RNG , class StartTransitionCallback , class SampleRecorder , class DiagnosticRecorder , class MessageRecorder >

|  |  |  |  |
| --- | --- | --- | --- |
| void stan::common::run\_markov\_chain | ( | stan::mcmc::base\_mcmc \* | *sampler*, |
|  |  | const int | *num\_iterations*, |
|  |  | const int | *start*, |
|  |  | const int | *finish*, |
|  |  | const int | *num\_thin*, |
|  |  | const int | *refresh*, |
|  |  | const bool | *save*, |
|  |  | const bool | *warmup*, |
|  |  | stan::io::mcmc\_writer< Model, SampleRecorder, DiagnosticRecorder, MessageRecorder > & | *writer*, |
|  |  | stan::mcmc::sample & | *init\_s*, |
|  |  | Model & | *model*, |
|  |  | RNG & | *base\_rng*, |
|  |  | const std::string & | *prefix*, |
|  |  | const std::string & | *suffix*, |
|  |  | std::ostream & | *o*, |
|  |  | StartTransitionCallback & | *callback* |
|  | ) |  |  |

Definition at line 13 of file run\_markov\_chain.hpp.

template<class Model , class RNG , class StartTransitionCallback , class SampleRecorder , class DiagnosticRecorder , class MessageRecorder >

|  |  |  |  |
| --- | --- | --- | --- |
| void stan::common::sample | ( | stan::mcmc::base\_mcmc \* | *sampler*, |
|  |  | int | *num\_warmup*, |
|  |  | int | *num\_samples*, |
|  |  | int | *num\_thin*, |
|  |  | int | *refresh*, |
|  |  | bool | *save*, |
|  |  | stan::io::mcmc\_writer< Model, SampleRecorder, DiagnosticRecorder, MessageRecorder > & | *writer*, |
|  |  | stan::mcmc::sample & | *init\_s*, |
|  |  | Model & | *model*, |
|  |  | RNG & | *base\_rng*, |
|  |  | const std::string & | *prefix*, |
|  |  | const std::string & | *suffix*, |
|  |  | std::ostream & | *o*, |
|  |  | StartTransitionCallback & | *callback* |
|  | ) |  |  |

Definition at line 13 of file sample.hpp.

template<class Model , class RNG , class StartTransitionCallback , class SampleRecorder , class DiagnosticRecorder , class MessageRecorder >

|  |  |  |  |
| --- | --- | --- | --- |
| void stan::common::warmup | ( | stan::mcmc::base\_mcmc \* | *sampler*, |
|  |  | int | *num\_warmup*, |
|  |  | int | *num\_samples*, |
|  |  | int | *num\_thin*, |
|  |  | int | *refresh*, |
|  |  | bool | *save*, |
|  |  | stan::io::mcmc\_writer< Model, SampleRecorder, DiagnosticRecorder, MessageRecorder > & | *writer*, |
|  |  | stan::mcmc::sample & | *init\_s*, |
|  |  | Model & | *model*, |
|  |  | RNG & | *base\_rng*, |
|  |  | const std::string & | *prefix*, |
|  |  | const std::string & | *suffix*, |
|  |  | std::ostream & | *o*, |
|  |  | StartTransitionCallback & | *callback* |
|  | ) |  |  |

Definition at line 13 of file warmup.hpp.

|  |  |  |  |
| --- | --- | --- | --- |
| void stan::common::write\_error\_msg | ( | std::ostream \* | *error\_stream*, |
|  |  | const std::exception & | *e* |
|  | ) |  |  |

Definition at line 11 of file write\_error\_msg.hpp.

template<class Model , class RNG >

|  |  |  |  |
| --- | --- | --- | --- |
| void stan::common::write\_iteration | ( | std::ostream & | *output\_stream*, |
|  |  | Model & | *model*, |
|  |  | RNG & | *base\_rng*, |
|  |  | double | *lp*, |
|  |  | std::vector< double > & | *cont\_vector*, |
|  |  | std::vector< int > & | *disc\_vector* |
|  | ) |  |  |

Definition at line 19 of file write\_iteration.hpp.

|  |  |  |  |
| --- | --- | --- | --- |
| void stan::common::write\_iteration\_csv | ( | std::ostream & | *output\_stream*, |
|  |  | const double | *lp*, |
|  |  | const std::vector< double > & | *model\_values* |
|  | ) |  |  |

Definition at line 10 of file write\_iteration\_csv.hpp.

|  |  |  |  |
| --- | --- | --- | --- |
| void stan::common::write\_model | ( | std::ostream \* | *s*, |
|  |  | const std::string | *model\_name*, |
|  |  | const std::string | *prefix* = `""` |
|  | ) |  |  |

Definition at line 11 of file write\_model.hpp.

|  |  |  |  |
| --- | --- | --- | --- |
| void stan::common::write\_stan | ( | std::ostream \* | *s*, |
|  |  | const std::string | *prefix* = `""` |
|  | ) |  |  |

Definition at line 12 of file write\_stan.hpp.


---

[ Stan Home Page ]

*© 2011–2014,
Stan Development Team.*
